# A Placebo-Controlled Study on the Treatment of Metabolic Syndrome of Qi Stagnation and Dampness Obstruction Related to Atypical Antipsychotics with Traditional Chinese Medicine (TCM)

**DOI:** 10.1155/2020/5103046

**Published:** 2020-07-30

**Authors:** Fengli Sun, Zhibin Ren, Yonghong Jiang, Xiangming Fang, Naixin Wang, Weidong Jin

**Affiliations:** ^1^Zhejiang Province Mental Health Center, Department of Psychiatry, Tongde Hospital of Zhejiang Province, Hangzhou, Zhejiang 310012, China; ^2^The Third Hospital of Huzhou City, Huzhou, Zhejiang 313000, China; ^3^Yiwu Mental Health Center, Yiwu, Zhejiang 322000, China; ^4^Department of Psychiatry, Cancer Hospital Affiliated to Chinese Academy of Medical Sciences, Zhejiang Cancer Hospital, Hangzhou, Zhejiang 310000, China; ^5^Zhejiang Chinese Medical University, Hangzhou, Zhejiang 311200, China

## Abstract

**Methods:**

154 schizophrenics who met both the diagnostic criteria of metabolic syndrome and qi stagnation and dampness obstruction syndrome were randomly divided into 2 : 1 groups. The PANSS and Tess were assessed before treatment and at the end of first month, second month, and third month after treatment; blood pressure, weight, waist circumference, hip circumference, blood glucose, glycosylated hemoglobin, high-density lipoprotein, low-density lipoprotein, triglyceride, and cholesterol were also measured at the same four time points. On the basis of continuous antipsychotic treatment, the study group took Liuyu decoction, and the control group took placebo.

**Results:**

Of the 154 cases, 102 were in the study group and 52 in the control group. Before and after treatment, there was a slight increase but no significant difference in blood pressure, blood sugar, glycosylated hemoglobin, cholesterol, TG, DHL, and LHL in two groups (*P* > 0.05) and also between the two groups (*P* > 0.05). The body weight, waist circumference, hip circumference, and BMI in the study group decreased, while that in the control group increased from the dividing group to the end of study. At the end of the third month, there was a significant difference in the body weight, waist circumference, hip circumference, and BMI between the two groups (*P* < 0.05). Before and after treatment, there was a significant difference in positive symptoms, negative symptoms, general symptoms, and PANSS in two groups, respectively (*P* < 0.05). The negative symptoms, general symptoms, PANSS, and TESS in the study group were lighter than that in the control group after treatment.

**Conclusion:**

Liuyu decoction is not only beneficial to the treatment in body constitution of metabolic syndrome in qi stagnation and dampness obstruction but also beneficial to the improvement of such patients' mental symptoms and side effects.

## 1. Background

Schizophrenia and other psychotic disorders are associated with high morbidity and mortality due to inherent health factors, genetic factors, and factors related to psychopharmacological treatment. Antipsychotics, like other drugs, have side effects that can substantially affect the physical health of patients, with substantive negative action on metabolism. By a prospective study, with a 24-month follow-up, Franch Pato et al. found that baseline abdominal circumference (lambda Wilks *P* < 0.01) and baseline HDL-cholesterol levels (lambda Wilks *P* < 0.01) were the parameters that seem to be more relevant than the rest of the metabolic syndrome constituents diagnosis criteria as predictors in the long-term, which seem that metabolic syndrome has nothing to do with atypical antipsychotics [1]. There seems to be some research studies that support this view. In an Italian sample, Ilaria Santini et al. report a metabolic syndrome prevalence of 27.5% in 389 subjects with psychotics. This figure is very close to the metabolic syndrome prevalence in the Italian general population quoted around 26% [2]. This is mainly due to the deleterious lifestyles characterized by physical inactivity, excessive alcohol consumption, smoking, and unhealthy diets common among patients with some psychotic disorders as well as due to cardiometabolic effects of psychotropic medications [3]. But many studies and results do not support this view.

Gül Dikeç et al. found that 73.8% of patients stated that they experienced side effects from antipsychotic medications, and 20.7% of patients experienced weight gain among 271 inpatients using atypical antipsychotic medications in a psychiatric hospital in Turkey [4]. Osama et al. found that after six months of taking the SGA, 44% of patients experienced elevated systolic pressure, 54.9% had elevated triglyceride, and 31.9% had impaired glucose levels (*P* value < 0.05). Prior to initiating SGA therapy, 14.3% of patients had metabolic syndrome, while 37.4% had metabolic syndrome after six months of therapy, and it was more prominent in males compared to female patients (*P* value < 0.05) [5]. Omer Saatcioglu et al. also found that the frequency of MS was 42.2% among the patients according to IDF (international diabetes federation) criteria. There was no significant difference between patients with and without MS in terms of age. The ratios of MS were 62.5% for the group taking typical and atypical antipsychotics together and 35.7% for the group taking two or more atypical antipsychotics together [6]. In China, the prevalence of metabolic syndrome in inpatients with schizophrenia was about 30% [7–9]. Antipsychotics are the most important treatment for schizophrenia and other mental disorders. At present, atypical antipsychotics, especially clozapine and olanzapine, which are widely used in clinic, can cause weight gain and greatly increase the risk of metabolic syndrome, type 2 diabetes, and cardiovascular disease. The incidence of metabolic syndrome is 2-3 times than that of the general population [9]. The atypical antipsychotics are different in the prevalence of metabolic syndrome. The clozapine greatly increases the risk of metabolic syndrome, and the prevalence was about 40%, while 35% for quetiapine, 30% for olanzapine [7], 20% for risperidone, 10% for aripiride, and 7% for ziprasidone [8]. So many methods for prevention and treatment of metabolic syndrome related to atypical antipsychotics in inpatients were taken in China [10]. Traditional Chinese Medicine (TCM) was one of the common ways for management of metabolic syndrome related to atypical antipsychotics [10].

The treatment of metabolic syndrome in TCM is based on syndrome differentiation. According to TCM, metabolic syndrome can be divided into spleen deficiency and dampness obstruction, phlegm and blood stasis, qi stagnation and dampness obstruction, and qi-yin deficiency [11]. Therapy decoction or method of these different syndromes of TCM is different. After the treatment of atypical antipsychotic drug-related metabolic syndrome with TCM decoction, body mass index, cholesterol, triglyceride, and low-density lipoprotein all decreased significantly [12]. Acupuncture can also reduce blood lipid, blood sugar, and waist circumference, and even diastolic pressure [13]. In clinical practice, the acupuncture and decoction were common for therapy of metabolic syndrome related to atypical antipsychotics. But these results need to be tested again, and the trials need to be compared to control or design as double-blind, multicenter. This study was taken blindly with placebo.

## 2. Materials

### 2.1. Diagnostic Criteria

The following patients were included: (1) the patients who meet the ICD-10 diagnostic criteria for schizophrenia [8]; (2) the patients who meet the diagnostic criteria for clinical metabolic syndrome [9]; (3) the patients who meet the diagnostic criteria for Qi Stagnation and Dampness Obstruction in TCM syndrome type [10]; (4) the PANSS of patients greater than or equal to 60 points; (5) the age of patients greater than or equal to 18 years old and less than or equal to 60 years old; (6) the patients taking atypical antipsychotics for 3 months or longer time; (7) the patients whose guardian informed consent of trials.

### 2.2. Exclusion Criteria

The following were the exclusion criteria: (1) brain organic diseases and mental disorders caused by them; (2) dependence on psychoactive substances and mental disorders caused by them; (3) some diseases for the presence and taking of hormones.; (4) Pregnant or lactating women; (5) metabolic syndrome before mental illness or taking atypical antipsychotic drugs; (6) the patients or their guardian who refused to this trial.

### 2.3. General Data

173 cases were collected, and 154 cases completed trials. 19 cases dropped out trial. Of the 19 patients who dropped off, 8 refused to take the decoction and quit the experiment, 6 quit the experiment due to the side effects of the original antipsychotic drugs, and 3 lost information due to discharge. Of 154 cases who completed trials, there were 87 males and 67 females. Their age was 18–60 years old. The average age was 45 ± 16 years. Height was 145–185 cm, with an average of 167 ± 13 cm. The course of disease was 0.8–15 years. The atypical antipsychotics taken include clozapine, olanzapine, risperidone, quetiapine, or combination of two drugs.

## 3. Methods

### 3.1. Randomization

The study group and the control group were randomly divided into 2 : 1 groups. The random method was divided into 2 : 1 groups according to the random table. There were 102 cases in the study group and 52 cases in the control group.

### 3.2. Assessment, Measurement, and Test

PANSS and Tess were evaluated before treatment and at the end of 1^st^, 2^nd^, and 3^rd^ month after treatment, respectively. The blood pressure, weight, waist circumference, and hip circumference were measured, respectively, at the same 4 time point. The fasting blood glucose, glycosylated hemoglobin, high-density lipoprotein, low-density lipoprotein, triglyceride, and cholesterol were also tested, respectively, at the same 4 time point.

The PANSS and TESS were assessed by two psychiatrists with medium-degree or high-degree professional title at least. The pair of psychiatrists has better reliability in assessment of PANSS and TESS.

Both the study and control groups took same supplements, other foods, and meals. There were same lifestyles such as exercise and food intake during the study duration between the groups, who were inpatients. And both the study and control groups took continuously their original antipsychotic medicine.

### 3.3. Treatment Method

While continuing to use atypical antipsychotic drugs, the subjects were randomly given TCM decoction or placebo. In the study group, the patients were given Liuyu decoction, which was composed as *Pinellia ternate*, *Gardenia, Cyperus, Fructus Aurantii, Atractylodes macrocephala, Magnolia officinalis*, and tangerine peel 12 g, respectively, Sichuan dome, *Poria cocos*, Amomum kernel 15 g, respectively, and licorice 6 g. Add 500 ml water and fry for 30 minutes; take 100 ml juice and take it orally twice, respectively, one dose per day for 3 months.

### 3.4. Preparation and Administration of Placebo

The placebo is prepared with brown pigment; the outer package and the color of the decoction are the same as that of the TCM decoction, but there is no taste of Chinese herbal medicine. Take 100 ml every morning and afternoon, same as TCM decoction.

### 3.5. Statistical Processing

All data were processed by SPSS18.0 statistical software, and the measurement data between groups were tested by the mean *t* test, and *P* < 0.05 was statistically significant.

### 3.6. Schedule of Trial

**Figure 1:**
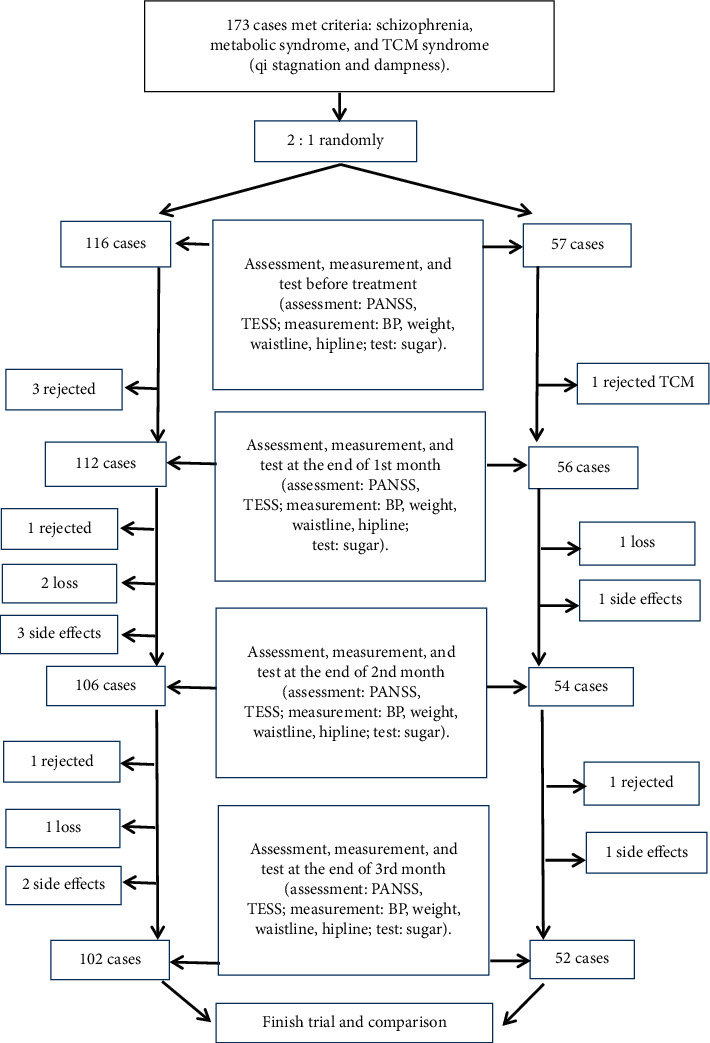
Schedule of trial.

The schedule for the trial performed is shown in Figure 1.

## 4. Results

### 4.1. Comparison of Blood Pressure and Body Constitution between Two Groups

Before and after the treatment, there was no significant difference in blood pressure between the two groups (*P* > 0.05). The body weight in the study group decreased while that in the control group increased. At the end of the third month, there was a significant difference in the body weight between the two groups. The body weight in the study group was significantly lighter than that in the control group (*P* < 0.05). Similar phenomena were also found in waist circumference, hip circumference, and BMI (Table 1).

### 4.2. Comparison of Blood Sugar, Glycosylated Hemoglobin, and Blood Lipids between Two Groups

Before and after treatment, there was no significant difference in blood sugar, blood sugar, glycosylated hemoglobin, cholesterol, TG, DHL, and LHL in two groups (*P* > 0.05) and also between the two groups (*P* > 0.05) (Tables 2 and 3).

### 4.3. Comparison of PANSS and TESS between Two Groups

Before and after treatment, there was significant difference in positive symptoms, negative symptoms, general symptoms, and PANSS in two groups, respectively (*P* < 0.05). But positive symptom in the study group was higher than that in the control group (*P* < 0.05), while this difference disappeared after treatment between two groups (*P* < 0.05). The negative symptoms, general symptoms, and PANSS in the study group were lighter than that in the control group after treatment, which suggest that Liuyu decoction may be effective in psychological symptoms (Table 4).

### 4.4. Comparison of TESS between Two Groups

Before and after treatment, there was a significant decrease in TESS in two groups, respectively (*P* < 0.05). And TESS in the study group was also lighter than that in the control group at the end of third month after treatment, which also suggests that Liuyu decoction may be beneficial in side effects of atypical antipsychotics (Table 5).

## 5. Discussion

The differential prevalence of metabolic syndrome associated with various atypical antipsychotic medications has been evidenced across numerous studies, with higher effects seen for certain antipsychotic medications on weight gain, waist circumference, fasting triglyceride level, and glucose levels [14]. Given the association of these symptoms, all atypical antipsychotic medications currently include a warning about the risk of hyperglycemia and diabetes, as well as suggestions for regular monitoring. The treatment of schizophrenia involves a balance in terms of risks and benefits. Failing to treat because of risk for complications from metabolic syndrome may place the patient at a higher risk for more serious health outcomes. It has been found that second generation antipsychotics (SGAs) are associated with weight gain, dyslipidaemia, and other metabolic derangements. The most important and first line of treatment for the metabolic syndrome is lifestyle changes including diet and exercise. However, other approaches such as the use of medication (e.g. metformin) have been also used, mainly when the lifestyle changes are difficult to achieve [15]. Metformin has been reported to counteract effectively antipsychotic-induced body weight gain and has been demonstrated to improve glycaemic control and promote a moderate weight loss in both diabetic and nondiabetic subjects. Metformin use appears to be a benefit when started early in the course of treatment and mostly in young adults newly exposed to antipsychotic drugs [15]. But drug interactions and increased economic costs are inevitable. The TCM may play an important role. In fact, clinical practice and research in this field have been carried out in China [10–13].

Chinese medicine has a long history of understanding metabolic syndrome. So far, a relatively perfect system has been formed from the etiology, pathogenesis, to clinics, such as TCM syndrome classification, syndrome standard criteria, and diagnostic differentiation and treatment [16]. The TCM pathological factors of metabolic syndrome are phlegm stagnation, blood stasis, and dryness and heat. The viscera involved are the liver, spleen, and kidney, and the phlegm stagnation and deficiency of the spleen is the root [17]. Deficiency of both the spleen and kidney results in endogenesis of water dampness, which induces stagnation of phlegm and continues to cause both stagnation of phlegm and blood [18]. This is the stagnation of qi and dampness that causes metabolic syndrome, and it is also a common TCM subtype or syndrome of metabolic syndrome [11, 17, 18]. In fact, the TCM syndrome types of metabolic syndrome include qi stagnation and dampness obstruction, phlegm and blood stasis, qi deficiency and blood stasis, and qi-yin deficiency; the former two syndromes are more common in clinical practice. There are some other syndrome types in clinical that can also be seen, such as liver depression and spleen deficiency [11, 17–19]. For those with qi stagnation and dampness obstruction, the main treatment principles are regulating qi, removing dampness, and resolving phlegm [13, 16, 17].

With therapeutic principles and research design, 152 cases in the study group took Liuyu decoction on the basis of the original antipsychotic drugs, and 52 cases in the control group took placebo on the basis of the original antipsychotic drugs. After 3 months, the body weight, hip circumference, waist circumference, and BMI of the study group were significantly lower than those of the control group, while the biological indicators, such as blood glucose, glycosylated hemoglobin, and blood lipid, were not different, and there was no difference in blood pressure between the two groups. It is suggested that Liuyu decoction may only affect the body constitution of patients.

Liuyu decoction can relieve all kinds of depression and dominate the syndrome of phlegm depression. The function is to move qi and relieve depression, to dry dampness, and dissipate phlegm. It mainly treats phlegm depression. Liuyu decoction is composed of Rhizoma Cyperi, *Atractylodes macrocephala*, Shenqu, Fructus Gardeniae, Fructus Forsythiae, tangerine peel, *Fritillaria*, Fructus Aurantii, Rhizoma smilax, and Radix Glycyrrhizae. In Liuyu decoction, *Pinellia ternate* has the functions of moistening and benefiting water, strengthening the spleen and stomach, and calming the nerves; *Amomum villosum* has the functions of moistening and appetizing the stomach, warming the spleen, and stopping diarrhea; *Atractylodes macrocephala* has the functions of moistening and strengthening the spleen; Ttngerine peel has the functions of regulating the qi and reducing the adverse, regulating the stomach, and drying the dampness and phlegm; Sichuan dome has the functions of activating the blood and removing the stasis, expanding the tubes, and reducing the blood pressure. Liuyu decoction, a compound preparation, is made up of above herbals and plays a therapeutic role in coordination.

It is worth mentioning that Liuyu decoction has a synergistic therapeutic effect on mental symptoms. The results showed that the total scores of negative symptoms, common symptoms, and PANSS in the study group were lower than those in the control group after taking Liuyu decoction for 3 months, and this difference was statistically significant, which indicated that Liuyu decoction might have a certain therapeutic effect on some mental symptoms. From the perspective of TCM, most mental diseases are related to phlegm, liver depression, blood stasis, and dampness obstruction [20, 21]. These pathological mechanisms in TCM are also related to metabolic syndrome. Therefore, this is the explanation that Liuyu decoction can improve both metabolic syndrome and mental symptoms. This is consistent with our previous observation and research, suggesting that the combination of traditional Chinese medicine and Western medicine can strengthen the effect of Western medicine. The change of symptoms in the combination group was significant than that in the single west group in treatment of patients with mania [22].

Interestingly, after taking Liuyu decoction for 3 months, the side effects of the study group were significantly lower than that of the control group, which was not only a good phenomenon but also a thing worth exploring. Although it is difficult to explain accurately, but from the perspective of TCM, it is related to the detoxification of Chinese herbals [23, 24]. One of the important benefits of integrated Chinese and Western medicine is to reduce the side effects of Western medicine, which seems to have been verified in this study.

## 6. Conclusion

Liuyu decoction is not only beneficial to the treatment in body constitution of metabolic syndrome in qi stagnation and dampness obstruction but also beneficial to the improvement of such patients' mental symptoms and side effects.

## Figures and Tables

**Table 1:** Comparison of blood pressure and body constitution between two groups.

	Study				Control			*t*	*P*
SBP (mmHg)	BT	126.76	±	16.63	127.15	±	13.64	−0.16	0.88
	1^st^ ME	126.25	±	13.81	122.92	±	14.21	1.39	0.17
	2^nd^ ME	122.33	±	13.03	123.88	±	13.09	−0.70	0.49
	3^rd^ ME	120.34	±	11.69	122.44	±	13.68	−0.94	0.35

DBP (mmHg)	BT	81.14	±	11.15	78.40	±	9.73	1.57	0.12
	1^st^ ME	80.41	±	8.88	77.67	±	10.03	1.66	0.10
	2^nd^ ME	79.08	±	8.20	76.63	±	8.64	1.69	0.09
	3^rd^ ME	76.85	±	7.70	74.83	±	10.58	1.22	0.22

Weight (kg)	BT	70.56	±	11.40	72.13	±	11.92	−0.78	0.44
	1^st^ ME	70.17	±	11.28	71.87	±	11.68	−0.86	0.39
	2^nd^ ME	69.56	±	10.89	72.52	±	12.01	−1.49	0.14
	3^rd^ ME	68.01	±	10.82	73.99	±	11.86	−3.04	0.00

Waistline (mm)	BT	95.09	±	12.39	97.33	±	12.65	−1.05	0.30
	1^st^ ME	94.39	±	12.52	97.75	±	15.75	−1.34	0.18
	2^nd^ ME	93.20	±	12.27	96.72	±	12.73	−1.64	0.10
	3^rd^ ME	91.77	±	9.81	97.39	±	12.33	−2.86	0.01

Hipline (mm)	BT	98.56	±	10.70	98.56	±	8.92	0.00	1.00
	1^st^ ME	97.67	±	12.34	98.18	±	8.68	−0.30	0.77
	2^nd^ ME	96.70	±	10.55	98.31	±	8.82	−1.00	0.32
	3^rd^ ME	95.45	±	10.69	97.86	±	8.55	−1.51	0.13

BMI	BT	25.99	±	4.11	26.19	±	4.07	−0.29	0.77
	1^st^ ME	25.83	±	3.96	26.08	±	3.90	−0.37	0.71
	2^nd^ ME	25.60	±	3.76	26.29	±	3.85	−1.05	0.30
	3^rd^ ME	25.02	±	3.65	26.85	±	3.98	−2.78	0.01

SBP = systolic blood pressure; DBP = diastolic blood pressure; BMI = body mass index; BT = before treatment; and ME = month end.

**Table 2:** Comparison of blood sugar and glycosylated hemoglobin between two groups.

		Study			Control			*t*	*P*
BS (mmol/L)	BT	5.60	±	2.12	5.35	±	1.70	0.79	0.43
	1^st^ ME	5.68	±	1.36	5.36	±	1.45	1.31	0.19
	2^nd^ ME	5.38	±	1.23	5.33	±	1.39	0.19	0.85
	3^rd^ ME	5.23	±	1.09	5.28	±	1.57	−0.20	0.84

GHb (%)	BT	5.96	±	1.45	5.66	±	0.95	1.51	0.13
	1^st^ ME	6.02	±	1.10	5.68	±	1.15	1.77	0.08
	2^nd^ ME	5.87	±	1.23	5.72	±	1.13	0.73	0.47
	3^rd^ ME	5.53	±	1.16	5.43	±	1.37	0.49	0.63

BS = blood sugar; GHb = glycosylated hemoglobin; BT = before treatment; and ME = month end.

**Table 3:** Comparison of blood lipids between two groups.

	Study				Control			*t*	*P*
Chol (mmol/L)	BT	5.20	±	1.17	4.88	±	1.02	1.73	0.09
	1^st^ ME	4.88	±	1.06	4.81	±	0.95	0.40	0.69
	2^nd^ ME	4.77	±	0.97	4.95	±	1.04	−1.04	0.30
	3^rd^ ME	4.61	±	0.97	4.74	±	0.88	1.05	0.30

TG (mmol/L)	BT	2.20	±	1.14	2.10	±	1.78	0.40	0.69
	1^st^ ME	2.24	±	1.39	2.08	±	1.58	0.60	0.55
	2^nd^ ME	2.22	±	1.27	2.21	±	1.69	0.04	0.97
	3^rd^ ME	5.61	±	11.44	3.81	±	2.19	1.48	0.14

LDL (mmol/L)	BT	2.48	±	0.97	2.65	±	0.70	−1.24	0.22
	1^st^ ME	2.88	±	0.88	2.61	±	0.71	2.07	0.04
	2^nd^ ME	2.82	±	0.89	2.66	±	0.70	1.23	0.22
	3^rd^ ME	4.61	±	0.97	4.74	±	0.88	−0.81	0.42

HDL (mmol/L)	BT	1.14	±	0.46	1.07	±	0.30	1.22	0.23
	1^st^ ME	1.16	±	0.46	1.07	±	0.27	1.42	0.16
	2^nd^ ME	1.16	±	0.52	1.12	±	0.38	0.63	0.53
	3^rd^ ME	1.97	±	1.11	2.01	±	1.59	−0.17	0.86

Chol = cholesterol; TG = triglyceride; LDL = low-density lipoprotein; HDL = high-density lipoprotein BT = before treatment; and ME = month end.

**Table 4:** Comparison of PANSS between two groups.

	Study				Control			*t*	*P*
PS	BT	18.42	±	6.63	16.04	±	4.69	2.58	0.01
	1^st^ ME	15.26	±	4.99	14.71	±	4.44	0.70	0.49
	2^nd^ ME	13.30	±	4.52	13.71	±	4.19	−0.56	0.58
	3^rd^ ME	11.82	±	4.10	13.17	±	4.38	−1.85	0.07

NS	BT	20.06	±	7.77	21.69	±	7.16	−1.30	0.20
	1^st^ ME	18.24	±	7.16	20.98	±	6.95	−2.29	0.02
	2^nd^ ME	16.83	±	7.07	19.85	±	6.75	−2.58	0.01
	3^rd^ ME	16.25	±	9.92	19.62	±	6.88	−2.45	0.02

GS	BT	34.34	±	9.76	32.52	±	6.46	1.38	0.17
	1^st^ ME	29.22	±	7.18	30.21	±	5.34	−0.97	0.34
	2^nd^ ME	27.05	±	6.58	29.71	±	5.45	−2.66	0.01
	3^rd^ ME	24.34	±	5.78	28.65	±	5.32	−4.61	0.00

PANSS	BT	72.82	±	19.18	70.25	±	12.68	0.99	0.32
	1^st^ ME	62.79	±	14.40	65.90	±	12.65	−1.37	0.17
	2^nd^ ME	57.33	±	13.83	63.27	±	13.07	−2.61	0.01
	3^rd^ ME	52.40	±	15.23	61.44	±	13.33	−3.79	0.00

PANSS = positive and negative symptom scale; PS = positive symptom; NS = negative symptom; GS = general symptom; BT = before treatment; and ME = month end.

**Table 5:** Comparison of TESS between two groups.

	Study				Control			*t*	*P*
TESS	BT	4.43	±	4.46	3.98	±	3.33	0.71	0.48
	1^st^ ME	3.93	±	3.51	3.69	±	2.63	0.47	0.64
	2^nd^ ME	3.38	±	3.09	3.63	±	2.61	−0.53	0.60
	3^rd^ ME	2.42	±	2.46	3.27	±	2.46	−2.02	0.05

TESS = treatment emergent symptoms scale; BT = before treatment; and ME = month end.

## Data Availability

The data used to support the findings of this study are available from the corresponding author upon request.

## Abbreviation

SPSS: Statistic product and service solutions
SBP: Systolic blood pressure
DBP: Diastolic blood pressure
BMI: Body mass index
BT: Before treatment
ME: Month end
BS: Blood sugar
GHb: Glycosylated hemoglobin
Chol: Cholesterol
TG: Triglyceride
LDL: Low-density lipoprotein
HDL: High-density lipoprotein
PANSS: Positive and negative symptom scale
PS: Positive symptom
NS: Negative symptom
GS: General symptom
TESS: Treatment emergent symptoms scale
SGA: Second generation antipsychotic.
